# The combination of radiomics features and VASARI standard to predict glioma grade

**DOI:** 10.3389/fonc.2023.1083216

**Published:** 2023-03-22

**Authors:** Wei You, Yitao Mao, Xiao Jiao, Dongcui Wang, Jianling Liu, Peng Lei, Weihua Liao

**Affiliations:** ^1^ Department of Radiology, Xiangya Hospital, Central South University, Changsha, China; ^2^ National Engineering Research Center of Personalized Diagnostic and Therapeutic Technology, Xiangya Hospital, Central South University, Changsha, China; ^3^ National Clinical Research Center for Geriatric Disorders, Xiangya Hospital, Central South University, Changsha, China; ^4^ Molecular Imaging Research Center, Central South University, Changsha, China

**Keywords:** nomogram, glioma grade, radiomics, VASARI, high-grade glioma, pre-operation diagnosis

## Abstract

**Background and Purpose:**

Radiomics features and The Visually AcceSAble Rembrandt Images (VASARI) standard appear to be quantitative and qualitative evaluations utilized to determine glioma grade. This study developed a preoperative model to predict glioma grade and improve the efficacy of clinical strategies by combining these two assessment methods.

**Materials and Methods:**

Patients diagnosed with glioma between March 2017 and September 2018 who underwent surgery and histopathology were enrolled in this study. A total of 3840 radiomic features were calculated; however, using the least absolute shrinkage and selection operator (LASSO) method, only 16 features were chosen to generate a radiomic signature. Three predictive models were developed using radiomic features and VASARI standard. The performance and validity of models were evaluated using decision curve analysis and 10-fold nested cross-validation.

**Results:**

Our study included 102 patients: 35 with low-grade glioma (LGG) and 67 with high-grade glioma (HGG). Model 1 utilized both radiomics and the VASARI standard, which included radiomic signatures, proportion of edema, and deep white matter invasion. Models 2 and 3 were constructed with radiomics or VASARI, respectively, with an area under the receiver operating characteristic curve (AUC) of 0.937 and 0.831, respectively, which was less than that of Model 1, with an AUC of 0.966.

**Conclusion:**

The combination of radiomics features and the VASARI standard is a robust model for predicting glioma grades.

## Introduction

Glioma, one of the most common central nervous system tumors (CNS), has a five-year survival rate of less than 5% and is widely recognized as a highly malignant tumor (1, 2). According to the WHO classification of brain tumors, pathology and clinical practices typically divide gliomas into low and high grades (3, 4). The prognosis of glioma patients would be improved by timely and accurate preoperative diagnosis (2). The primary treatment for gliomas is surgical resection followed by radiotherapy or chemotherapy (5). As a non-invasive technique, MRI will likely be utilized in the clinical setting to detect glioma and its clinical grade early and reduce misdiagnosis (6). When developing clinical strategies for patients suspected of having glioma, conventional MRI sequences, such as T1-weighted, contrast-enhanced T1-weighted MR images, T2-weighted, and fluid-attenuated inversion recovery (7) are frequently employed.

Recent neoplasia research (8–10) has extensively used radiomics as a promising method for evaluating tumor characteristics. This semiautomatic method can quantify the high-dimensional imaging features of glioma by extracting the radiomic features from conventional medical images and combining these features with other clinical information to design a machine-learning model, which will improve the accuracy and efficiency of clinical decisions (11–14). In contrast to the tumor phenotype and microenvironment provided by clinical reports and histopathology, this information is based on intensity, shape, size, volume, and texture (15). Park et al. (16) extracted radiomic features from multiparametric MRI to predict LGGs and a subgroup of LGGs without enhancement. In the internal validation set, the area under the receiver operating characteristic curve (AUC) was 0.85 and 0.82, indicating the best performance. Mao et al. (17) predicted glioma grade using an artificial neural network model based on image data. The model had a means accuracy of 90.32%, sensitivity of 87.86%, and specificity of 92.49%. With the exponential growth of medical image analysis, radiomics is increasingly used to detect cancer, evaluate prognosis and treatment, and monitor tumor status. Glioma grade has been the subject of much research; however, it is still of utmost importance because of its relevance to clinical treatment and pre-surgical strategies.

Visually AcceSAble Rembrandt Images (VASARI) features of glioma have 25 qualitative features for human gliomas in particular (18). On standard pre- and post-contrast-enhanced MRI, these features represent common characteristics of primary cerebral neoplasia and are described using standardized terminology. Chen et al. (19) combined radiomics with qualitative features (VASARI annotations and T2-FLAIR mismatch signs) to predict molecular subtypes in patients with lower-grade glioma. The AUC of the model containing radiomics and qualitative features was higher than the AUC of the model containing radiomics alone, with 0.8623 versus 0.6557. Cao et al. (20) demonstrated that the AUC of the IDH1 mutation predictive model with VASARI features alone was approximately 0.827 in the training group; however, in the fusion model with optimal VASARI and radiomics features, the AUC improved to 0.879, with an accuracy of 0.771, exceeding that of the model with VASARI alone (approximately 0.726). Therefore, a fusion model combining radiomics and VASARI features would better predict glioma grade than either model alone.

Our study aimed to determine the impact of VASARI features on the basics of radiomics and whether the introduction of VASARI features adds predictive value to glioma grade. The research was conducted exclusively at Xiangya Hospital.

## Materials and methods

### Patients

**Figure 1:**
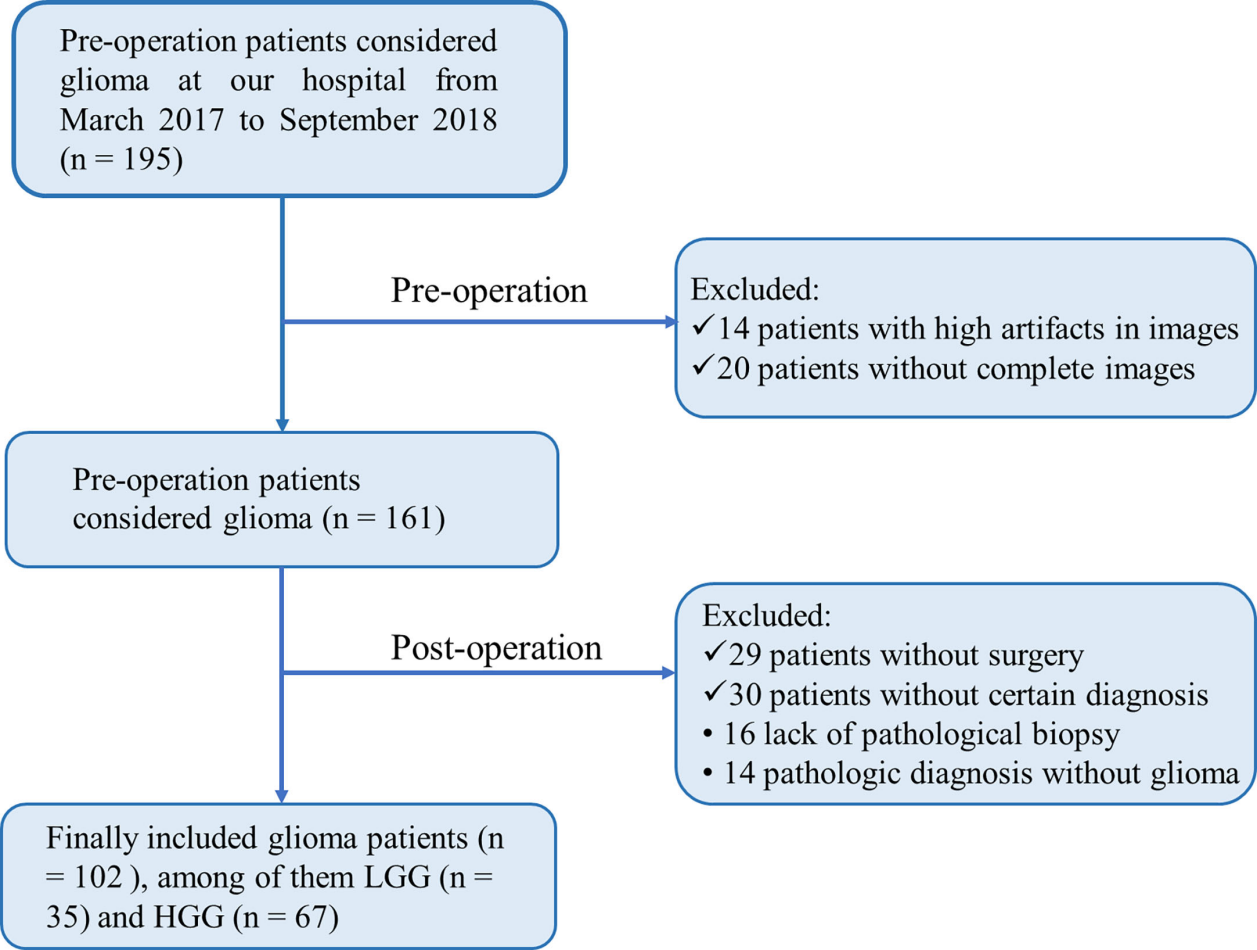
Flowchart of inclusion and exclusion process for patients to be enrolled.

The Medical Ethics Committee of our institution provided Ethical approval, followed by the informed consent principle. From March 2017 to September 2018, 102 patients who met the following criteria were enrolled in this study: pathologic diagnosis of glioma without prior treatment and MR data free of severe artifacts. Medical records were extracted from an institutional database. Our institutional Ethics Committee and Review Board approved this retrospective study. Written informed consent was waived owing to the retrospective nature of this investigation. Additional information regarding the patient recruitment procedure and exclusion criteria is presented in **Figure 1**.

### Pathological re-assessment

The paraffin-embedded surgical specimens were re-assessed by two experienced pathologists at our institution (with over 10 and 15 years of experience, respectively) in tumor imaging diagnosis of the central nervous system (CNS), using the 2021 WHO classification of CNS tumors (4).

### Image acquisition

Untreated glioma patients underwent MRI using a 3.0-T imaging unit (Siemens, Erlangen, Germany) and a 64-channel receiver head coil. In the transverse plane, spoiled gradient-recalled images were used to acquire T1-weighted anatomic images (T1WI), T2-weighted anatomic images (T2WI), and fluid-attenuated inversion recovery (FLAIR). Dynamic gadodiamide (SFDA approval number J20100063, produced by GE Healthcare Ireland) high-resolution, three-dimensional magnetization-prepared rapid acquisition with gradient-echo sequences (3D-T1-MPRAGE) were used to perform 10 ml contrast material-enhanced MRI on patients.

### Image segmentation

Segmentation of regions of interest (ROI) was performed on the T1WI, T2WI, FLAIR, and contrast-enhanced 3D-T1-MPRAGE images. Using ITK-SNAP (21) (http://www.itksnap.org), two experienced radiologists (reader 1 and 2, with more than ten years of experience in neuroimaging) manually delineated the tumor boundaries slice-by-slice. The two radiologists were blinded to the patient information, including radiological and clinicopathological data. Fifty patients were randomly selected to evaluate the inter-observer (reader 1 versus reader 2) and intra-observer (reader 1 twice at intervals of four weeks) correlation coefficient (ICC). Generally, consistency was indicated by an ICC greater than 0.75. For the randomly selected 50 patients, the first segmentation of reader 1 was used. The ROI contouring work of the remaining patients was completed only by reader 1. The tumor ROIs were manually delineated on T1WI, T2WI, FLAIR, and contrast-enhanced 3D-T1-MPRAGE images, and only the axial direction was involved in ROI contouring.

### Radiomic feature selection

The radiomic features were extracted using PyRadiomics in Python (version. 3.7, https://www.python.org/). Extracted features included Shape, first-order intensity statistics, Gray Level Co-occurrence Matrix, Gray Level Size Zone Matrix, Gray Level Run Length Matrix, Gray Level Dependence Matrix, logarithm, and Wavelet. Features with ICC values less than or equal to 0.75 were supposed to be excluded from further analyses. Using the least absolute shrinkage and selection operator (LASSO) method (22), the most relevant radiomics features associated with glioma grading were determined. *Z*-score normalization was used as a preprocessing step for LASSO. Then, the weighted average method with the respective LASSO coefficients was used to linearly combine the most relevant features into a single index called radiomic signature (Rad-score). This Rad-score was deemed an independent variable, along with other image-related VASARI variables.

### Clinical feature selection

Univariate and multivariate logistic regression were used to select the most relevant predictors (including Rad-score and the VASARI features) for high-grade glioma, with a p-value of 0.10 (for univariate logistic regression) and 0.05 (for multivariate logistic regression) as the significance level, respectively. In this study, logistic regression was utilized because its outputs were probabilities, which allowed subsequent calibration analysis, nomogram plotting, and decision curve analysis, which are required to comprehensively assess the performance of a predictive model. Two neuroradiologists assessed all VASARI imaging features on standard pre- and post-contrast-enhanced MRI with 8 and 12 years of experience on the open-source picture archiving and communication system (PACS) workstation. Disagreements were addressed through discussions.

### Model assessment and validation

In this study, we assessed four aspects of a predictive model, *i.e.*, robustness, discrimination, accuracy, and clinical applicability. The robustness of the model was evaluated by 10-fold nested cross-validation (with an outer loop of ten folds for test cohorts and an inner loop of nine folds for training and validation cohorts). The folds in this cross-validation were stratified, that is, similarly distributed for the positive and negative samples.

The area under the curve (AUC) of the receiver operating characteristic (ROC) curves was used as the performance index. The standard deviation of the ten AUCs was then calculated to assess the robustness (stability) of the model. The discrimination performance of the model was evaluated solely based on the AUC itself. The calibration curve assessed the accuracy of the model, which indicated the degree of agreement between the observed probabilities and model-predicted probabilities using a bootstrap method (1000 resampling iterations). The Hosmer-Lemeshow test was conducted to determine if the level of agreement was statistically significant (23). Clinical applicability was evaluated using a decision curve analysis, which quantitatively suggested whether the model would result in a net benefit for those patients who use it in clinical practice compared to arbitrary decisions (*i.e.*, treat all patients or treat none) (24).

### Statistical analysis

**Figure 2:**
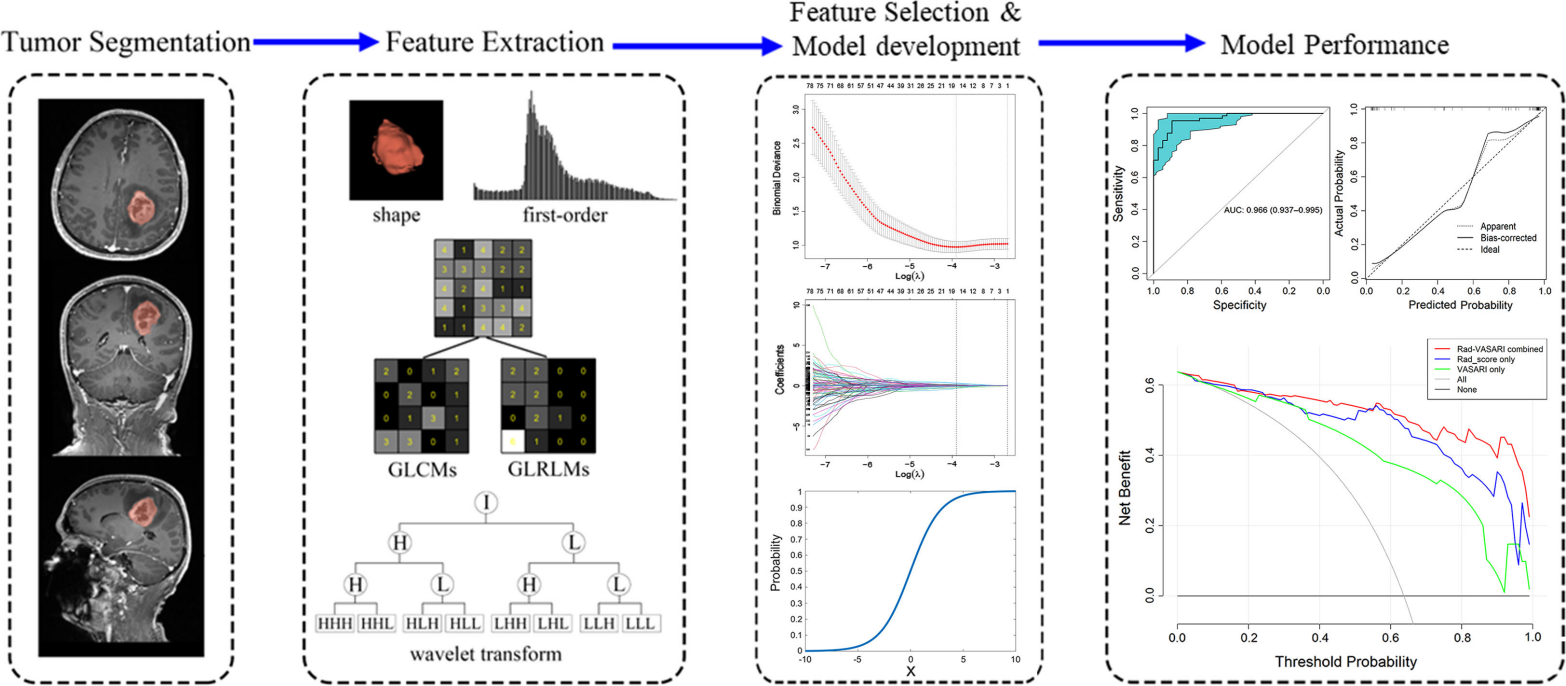
Workflow for the method section. Firstly, tumor segmentation was depicted on the MRI images. Secondly, five categories of radiomic features were extracted from the tumor, including shape, first-order, gray level co-occurrence matrix (GLCM), gray level run-length matrix (GLRLM), and wavelet transform. Thirdly, the least absolute shrinkage and selection operator (LASSO) method was used on feature selection, with model development shown. Finally, the ROC, calibration curve and decision curve analysis was used to assess the model performance.

All statistical analyses were conducted with R software version 4.0.2 (http://www.Rproject.org) using the following packages: “glmnet,” “rms,” “pROC,” “rmda,” and “broom.” The “glmnet” was used to execute the LASSO method. A nomogram was created using the “rms” function. The AUCs of different ROC curves were compared using the deLong test (25) in “pROC” package. Calibration was assessed using R software, with the “calibrate” function in R package “rms”. The Hosmer-Lemeshow test (23) was used to determine the significance of the calibration curve. All statistical tests were two-sided, and the *p*-value of statistical significance was set to 0.05, except for the univariate logistic regression used to screen out potential variables, for which the *p*-value was set to 0.10. The workflow of this study is illustrated in **Figure 2**.

## Results

### Patient characteristics

Our study included 102 patients, with 37 in LGG group (I/II 2/35) and 65 in HGG group (III/IV, 26/39). **Table 1** summarizes the VASARI features, Rad-score, and age and gender ratio for LGG and HGG groups.

**Table 1:** The results of binary logistic regression analysis for predictive models.

Factors	Univariate analysis			Multivariate analysis		
	OR	95% CI	*p* value	OR	95% CI	*p* value
**Gender**	0.957	0.688-1.331	0.882			
**Age**	1.080	0.659-1.770	0.908			
**VASARI**						
**F1**	1.036	0.857-1.252	0.922			
**F2**	1.541	0.926-2.565	0.465			
**F3**	0.916	0.645-1.301	0.787			
**F4**	1.255	0.831-1.895	0.652			
**F5**	0.946	0.722-1.240	0.897			
**F6**	1.178	0.885-1.568	0.736			
**F7**	0.933	0.691-1.260	0.807			
**F8**	2.036	1.029-4.029	0.046	1.136	0.986-1.309	0.088
**F9**	1.252	0.836-1.875	0.688			
**F10**	0.853	0.492-1.479	0.162			
**F11**	1.461	0.896-2.382	0.507			
**F12**	0.952	0.757-1.197	0.776			
**F13**	1.281	0.853-1.924	0.439			
**F14**	3.638	1.088-12.165	0.029	2.152	1.029-4.501	0.036
**F15**	1.109	0.905-1.371	0.786			
**F16**	1.266	0.932-1.720	0.409			
**F17**	0.899	0.673-1.201	0.533			
**F18**	1.426	0.811-2.507	0.427			
**F19**	1.058	0.957-1.170	0.456			
**F20**	1.436	0.913-2.259	0.221			
**F21**	3.895	1.120-13.546	0.011	2.487	1.094-5.654	0.026
**F22**	0.895	0.616-1.300	0.436			
**F23**	1.127	0.882-1.440	0.649			
**F24**	2.587	1.031-6.491	0.035	1.195	0.992-1.440	0.062
**F25**	1.225	0.894-1.679	0.587			
**Rad-score**	13.661	3.688-50.603	<0.001	18.604	4.257-81.303	<0.001

CI, confidence interval; OR, odds ratio. VASARI, The Visually AcceSAble Rembrandt Images. F1: Tumor Location. F2: Side of Tumor Epicenter, F3: Eloquent Brain. F4: Enhancement Quality. F5: Proportion Enhancing. F6: Proportion nCET. F7: Proportion Necrosis. F8: Cyst(s). F9: Multifocal or Multicentric. F10: T1/FLAIR RATIO. F11: Thickness of enhancing margin. F12: Definition of the enhancing margin. F13: Definition of the non -enhancing margin. F14: Proportion of Edema. F15: Edema Crosses Midline. F16: Hemorrhage. F17: Diffusion. F18: Pial invasion. F19: Ependymal invasion. F20: Cortical involvement. F21: Deep White Matter Invasion. F22: nCET tumor Crosses Midline. F23: Enhancing tumor Crosses Midline. F24: Satellites. F25: Calvarial remodeling.

### Radiomic feature extraction

A total of 3840 features from T1WI, T2WI, FLAIR, and contrast-enhanced 3D-T1-MPRAGE images were extracted using Pyradiomics, including shape (14 features), first-order intensity statistics (18 features), Gray Level Co-occurrence Matrix (22 features), Gray Level Size Zone Matrix (16 features), Gray Level Run Length Matrix (16 features), Gray Level Dependence Matrix (14 features), logarithm (172 features), and wavelet (688 features). All features had high ICCs (0.8491 0.9807). Using LASSO logistic regression on the entire cohort, only 16 features survived based on the minimum criterion; the remaining features were omitted because their coefficients were compressed to zero per the LASSO minimum criterion (**Figure 2**, Feature Selection). The remaining 16 features are listed in **Table 2**. The Rad-score is then calculated as the linear sum of these 16 non-zero coefficient weighted features (26).

**Table 2:** Selected radiomic features and its coefficients.

	Selected features	Coefficient
Contrast-enhanced 3D-T1-MPRAGE	original_firstorder_MeanAbsoluteDeviation	0.008376
	original_firstorder_Mean	-0.21765
	logsigma_3_0_mm_3D_glcm_Correlation	0.014965
	log_sigma_5_0_mm_3D_glszm_GrayLevelNonUniformityNormalized	0.163822
	log_sigma_5_0_mm_3D_glszm_SmallAreaLowGrayLevelEmphasis	0.354926
	wavelet_LHL_gldm_DependenceVariance	-0.02259
	wavelet_LHH_gldm_LargeDependenceHighGrayLevelEmphasis	0.061954
	wavelet_HLL_glcm_JointEntropy	-0.1377
	wavelet-LLL_gldm_SmallDependenceLowGrayLevelEmphasis	0.069576
T1WI	log-sigma-5-0-mm-3D_glcm_SumEntropy	0.176542
	wavelet-HHL_glcm_JointAverage	0.000975
T2WI	log-sigma-5-0-mm-3D_gldm_LowGrayLevelEmphasis	0.027959
	wavelet-LHH_glrlm_LongRunEmphasis	-0.26765
FLAIR	log-sigma-5-0-mm-3D_gldm_GrayLevelVariance	-0.036765
	wavelet-LHL_gldm_SmallDependenceLowGrayLevelEmphasis	-0.316588
	wavelet-HLL_firstorder_Kurtosis	0.117975

### Construction of predictive models

The results of univariate and multivariate logistic regression analyses are presented in **Table 1**. As final predictors, three variables remained: edema proportion, deep white matter invasion, and Rad-score. Note that the percentages of edema and deep white matter invasion are VASARI features. Based on the outcomes of logistic regression, three predictive models were developed. Model 1 was constructed with all three final predictors; Model 2 was constructed with Rad-score alone, and Model 3 was constructed with the remaining two VASARI variables after Rad-score was omitted.

### Model performance

#### Robustness

**Figure 3:**
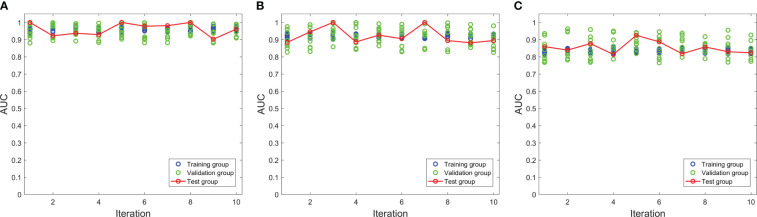
The performances of three models in the nested cross-validation. **(A–C)** represent model 1, model 2 and model 3 respectively.

The 10-fold nested cross-validation was performed to assess the performance stability of the model. **Figure 3** depicts the performance of the three models in 10-fold nested cross-validation. The ten iterations for the test cohort had standard deviations of 0.0362, 0.0458, and 0.0355 for models 1, 2, and 3, respectively. Thus, all three models were relatively stable throughout the ten repetitions in terms of AUC.

#### Discrimination

**Figure 4:**
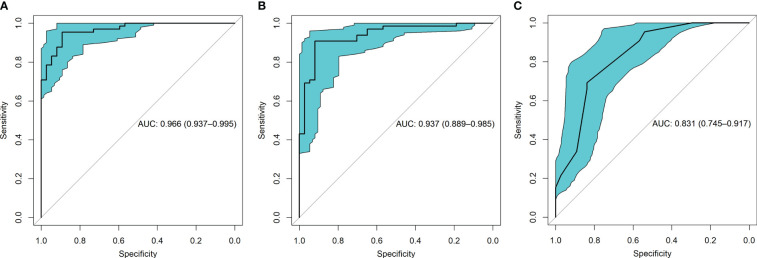
The receiver operating characteristic (ROC) curves for the three models were shown. **(A)** presented the ROC of model 1 which combined radiomic and VASARI features, with the area under the curve (AUC) of 0.966. **(B)** displayed the ROC of model 2 including radiomic alone, with the AUC of 0.937. **(C)** presented the ROC of model 3 including VASARI features alone, with the AUC of 0.831.

ROC curve indicates the discriminatory ability of a diagnostic/predictive model. **Figure 4** displays the ROC curve analyses of the three models. Model 1, Model 2, and Model 3 had AUCs of these ROC curves for predicting glioma grade of 0.966 (95% CI: 0.937–0.995), 0.937 (95% CI: 0.889–0.985), and 0.831 (95% CI: 0.745–0.917), respectively. **Table 3** displays the remaining indices of ROC curves, including the sensitivity, specificity, positive predictive value, negative predictive value, and accuracy.

**Table 3:** Performance of the three predictive models.

Metrics	Model 1 (Rad−VASARI combined)	Model 2 (Rad_score only)	Model 3 (VASARI only)
**AUC**	0.966	0.937	0.831
**Accuracy**	0.931	0.912	0.761
**Sensitivity**	0.954	0.908	0.776
**Specificity**	0.892	0.919	0.723
**PPV**	0.939	0.952	0.681
**NPV**	0.917	0.850	0.596

AUC: area under the receiver operating characteristic curve; PPV: positive predictive value; NPV: negative predictive value.

The AUC cut-off was determined based on Youden index maximization criterion. Specifically, Youden index = true positive rate (sensitivity) – false positive rate (1-specificity). In the ROC curve, a series of Youden indices was calculated, then the maximum Youden index of this series was picked out and the corresponding value of the test variable which matched this maximum Youden index was the cut-off value.

#### Accuracy

**Figure 5:**
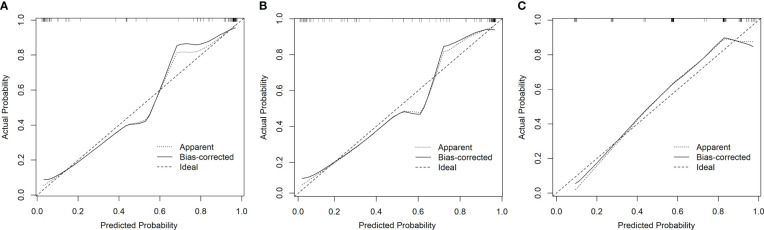
The calibration curves of the three models showed good consistency between the predicted probability of HGG and the observed probability of HGG (**A** model 1, **B** model 2, and **C** model 3).

The accuracy refers to the consistency between the predicted and observed values, which is reflected in the calibration curve. The calibration curves of the three models demonstrated a good agreement between the predicted and observed probabilities of HGG (**Figure 5**). All these curves failed to reach statistical significance according to the Hosmer-Lemeshow test (all p > 0.05), indicating that there is good agreement with the ideal diagonal line (i.e., good fitting between the predicted and the observed HGG probability). Because all three calibration curves were statistically well-fitted and exhibited no discernible deviation from the ideal line, we could not select the best-fitted curve.

#### Clinical applicability

**Figure 6:**
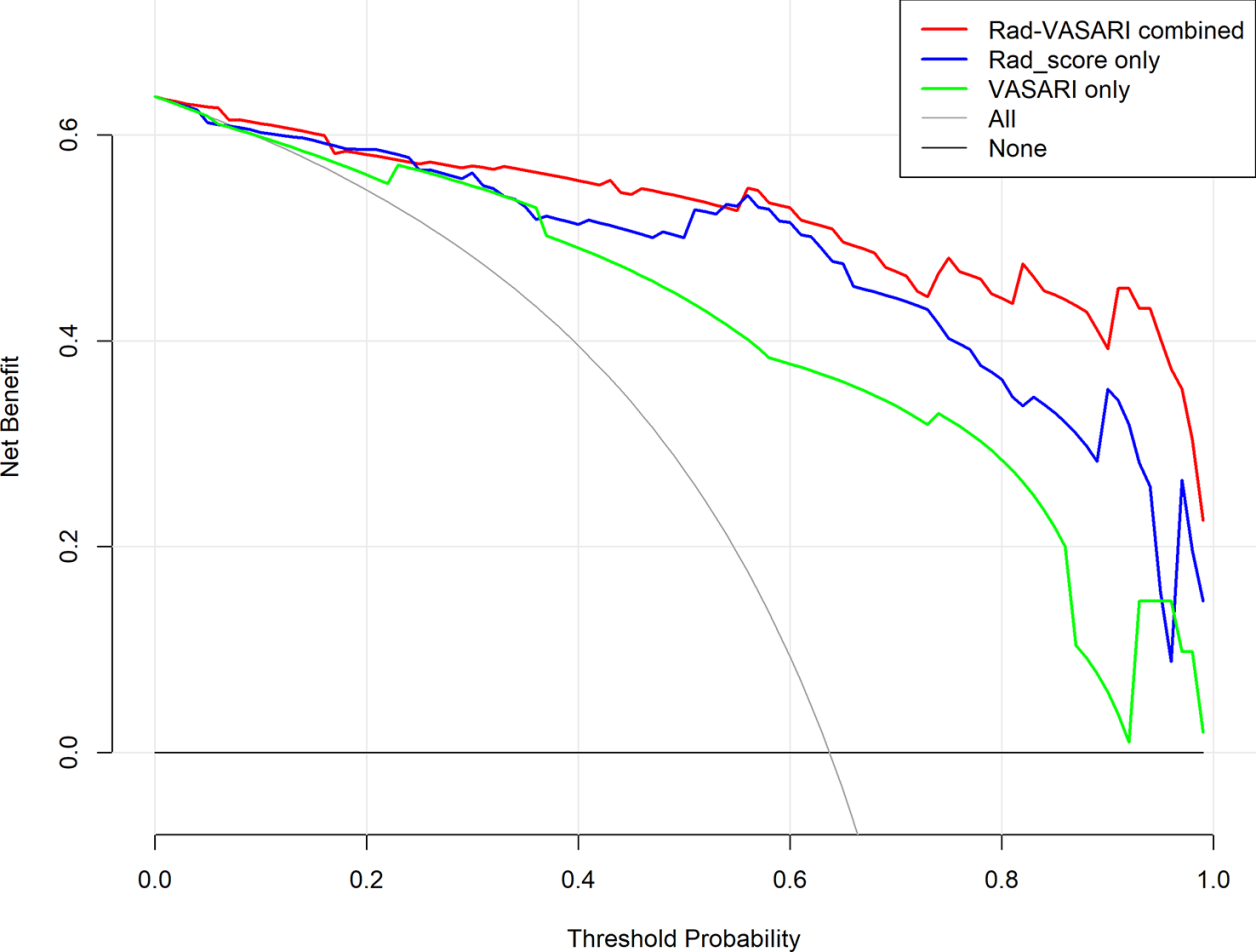
Decision curves for the three models. Red, combined radiomic and VASARI features model; blue, radiomic model; green, VASARI features model.

**Figure 7:**
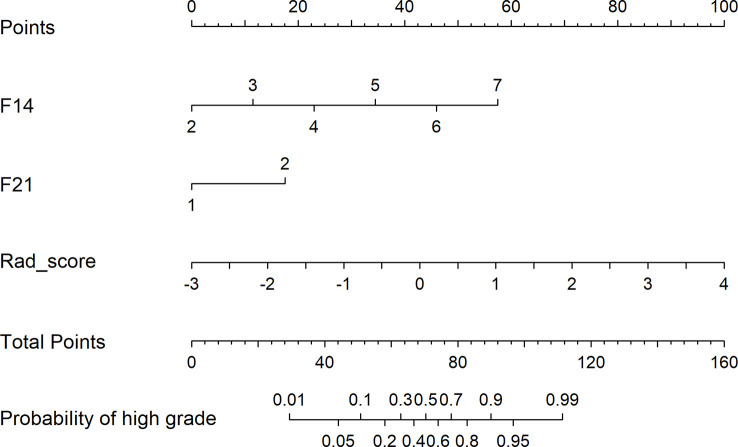
The nomogram of Model 1 combined radiomic and VASARI features.

**Figure 6** depicts the decision curves of the models. These models are separated from the “treat all” or “treat none” lines, indicating that they may have clinical utility. However, Model 1 appears to have the highest position, indicating that using Model 1 to grade glioma would provide patients with the greatest net benefit compared with Models 2 and 3. Regarding glioma grading, our results indicate that Model 1 (combining radiomics and VASARI variables) is the optimal model among the three models and could be the preferable model for regular clinical practice. **Figure 7** depicts the nomogram of model 1 to facilitate its clinical application.

## Discussion

In this study, we developed a predictive model for glioma grade before surgery and histopathology. This model, constructed using radiomics and two VASARI features, performed well in distinguishing LGG from HGG patients. The performance of the model was comprehensively evaluated based on its discrimination, calibration, and clinical utility. The 10-fold nested cross-validation also confirmed the stability and robustness of the model. In addition, our study suggests that radiomics and VASARI could be used to independently predict glioma grade.

With only 102 patients enrolled, the conventional method of dividing the samples into training and testing cohorts was insufficient to evaluate the robustness of our model. Our study evaluated the robustness of the predictive models using 10-fold cross-validation. There have been published radiomics studies with validation cohorts as small as 20–30 patients, making the performance of such models questionable owing to the risks of overfitting and high instability (27). Meanwhile, the external validation cohort sample size should ideally be between 25%–40% of the training cohort (27), although it is common for published studies to violate this requirement. Wang et al. (28) recruited 85 patients and divided them into a training cohort (n = 56) and a validation cohort (n = 29) to develop a radiomics nomogram for glioma grade prediction. The authors discovered that the radiomics nomogram had an excellent C-index of 0.971 in the training cohort and 0.961 in the validation cohort. Given the limited number of samples and the disparity between HGG and LGG, separating the data into training and validation datasets would further reduce the sample size, resulting in highly unstable performance. For a limited cohort, nested cross-validation could be a preferable method to assess whether the selected features are stable across the different folds and to avoid circularity bias while measuring prediction performance (29–31). Our study provided additional information on nested cross-validation from the dividing cohort to AUC scores, enhancing the credibility and confirming the robustness of our models by presenting a transparently detailed procedure.

Regarding the clinical features we selected, the proportion of edema and deep white matter invasion were two key indicators of the malignant behavior of glioma. First, the incidence of peritumoral edema (PTE) is significantly associated with glioma morbidity and mortality. According to previous studies, the average or overall survival of patients with significant edema (> 10 mm) was reduced by more than half compared to those with minor edema (32, 33). In a previous study, Wu et al. (33) hypothesized that edema shape resulting from the extent of edema also influences patient survival. Patients with an irregular edema shape (such as a radial or finger-like shape) tended to have a worse prognosis than those with round edema. In addition, Jeong et al. (34) found that amplification of the epidermal growth factor receptor (EGFR) plays a significant role in the formation of PTE and causes the volume of edema to increase, thereby negatively affecting overall survival. Some studies have indicated that HMGB1 suppression and LINC00665 expression are closely associated with PTE (35, 36).

The deep white matter invasion that we selected to represent malignant glioma was also significant in a previous study. Tumor location, a crucial parameter for patient care, correlates strongly with molecular subtypes, histopathological characteristics, clinical presentation and surgery, surgical management, glioma malignancy level, and prognosis (37–42). Roux et al. (38) presented probabilistic maps based on clinical presentations and survival analysis. Their results demonstrated that tumors in the deep location and eloquent brain regions were more likely to be associated with poor prognosis and shorter overall survival than those in the superficial location distant from the eloquent area.

Invasion along the white matter tracts is an important clinicopathological characteristic of gliomas, indicative of poor therapeutic prognosis (37, 43). Our study utilized the VASARI standard and combined it with contrast-enhanced 3D-T1-MPRAGE radiomics for analysis, which should be superior to using VASARI alone because radiomics analysis should be more objective, accurate, and reliable as a quantitative method. As a non-invasive diagnostic method, radiomic features extracted from images reflect cellular behaviors in the intratumoral microenvironment, which correlates with the prognosis of the tumor (44–46). Heterogeneity, an important parameter of the clinicopathological characteristics of gliomas, is associated with the degree of malignant behavior (47). For instance, tumors with more aggressive behavior may indicate higher heterogeneity, whereas tumors with more favorable behavior tend to exhibit less heterogeneity. Our study filtered kurtosis and entropy-related radiomic features using the LASSO method, indicating greater heterogeneity. According to a previous study (48), kurtosis and entropy are significant indicators of glioma heterogeneity. Spatial and temporal vascular anomalies, which result from hypoxia and acidosis within the tumor caused by angiogenesis, are primary contributors to tumor heterogeneity (47). The models in our study were consistent with those in previous research, suggesting that kurtosis and entropy reflect greater heterogeneity and a worse prognosis. Among the most relevant 16 radiomic features (**Table 2**), nine features were derived from contrast-enhanced 3D-T1-MPRAGE, two were derived from T1WI, two were derived from T2WI, and three were derived from FLAIR, indicating that 3D-T1-MPRAGE could be the essential sequence and exerted the largest contribution for identifying the glioma grade.

Our study had several limitations. First, the small sample size was insufficient to maintain the stability of the results. Therefore, we utilized nested cross-validation to confirm the validity of the predictive model. Second, our study lacked the molecular subtype for the samples, while the molecular phenotype is crucial for the prognosis of glioma (49, 50). Future medical imaging research should focus on the molecular characteristics of glioma, which could aid in more accurate subtype prediction and the development of individual treatment strategies.

## Conclusion

This study demonstrates the significance of a predictive model combining radiomics features with VASARI standard for glioma grade analysis before surgical intervention. This non-invasive imaging-centered strategy would aid in advancing clinical research and guiding individualized treatment for patients with high-grade glioma.

## Data availability statement

The raw data supporting the conclusions of this article will be made available by the authors, without undue reservation.

## Ethics statement

Written informed consent was obtained from the individual(s) for the publication of any potentially identifiable images or data included in this article.

## Author contributions

WY, YM, XJ, PL, and WL contributed to conception and design of the study. WY and XJ organized the database, finished image segmentation and evaluated clinical features. DW extracted radiomic features. JL was responsible to the data collection in prior. WY and YM processed the pictures and tables, and performed the statistical analysis. PL also joined the pictures processing. WY wrote the first draft of the manuscript. YM wrote sections of the manuscript. All authors contributed to manuscript revision, read, and approved the submitted version.
